# Association between rectal diameter and response to treatment with parasacral transcutaneous electrical nerve stimulation and behavioral changes in children and adolescents with bladder and bowel dysfunction

**DOI:** 10.1590/S1677-5538.IBJU.2023.0201

**Published:** 2024-02-07

**Authors:** Noel Charlles Nunes, Glicia Estevam de Abreu, Eneida Regis Dourado, Maria Luiza Veiga, Ananda Nacif, Maria Thaís de Andrade Calasans, Ana Aparecida Nascimento Martinelli Braga, Ubirajara Barroso

**Affiliations:** 1 Escola Bahiana de Medicina e Saúde Pública Centro de Distúrbios Urinários na Infância Salvador BA Brasil Centro de Distúrbios Urinários na Infância (CEDIMI), Escola Bahiana de Medicina e Saúde Pública, Salvador, BA, Brasil

**Keywords:** Lower Urinary Tract Symptoms, Pediatrics, Ultrasonography

## Abstract

**Purpose:**

Parasacral Transcutaneous Electrical Stimulation (TENS) is one of the treatments for children with Bladder and Bowel Dysfunction (BBD). Some studies showed that children with increased Rectal Diameter (RD) have more Functional Constipation (FC). However, RD prediction in maintenance of BBD after treatment was never evaluated. Our aim is to evaluate the association between RD and response to treatment in children and adolescents with BBD.

**Materials and Methods:**

This study evaluated patients from 5-17 years old with BBD. Dysfunctional Voiding Scoring System (DVSS), Rome IV criteria, and the Constipation Score were used. RD was measured using abdominal ultrasound before treatment according to the technique established by Klijn et al. and was considered enlarged when >3cm. No laxatives were used during treatment. Descriptive analysis and binary regression were performed and the area under the ROC curve was calculated.

**Results:**

Forty children were included (mean age 8.4±2.8 years, 52.5% male). Before treatment, RD was enlarged in 15 children (37.5%) (mean diameter 3.84±0.6cm), with FC persisting post-treatment in 11/15(73.3%). Those patients also required more laxatives following treatment and had more severe FC. Binary regression showed pretreatment RD to be an independent predictor of the persistence of FC post-treatment (OR=9.56; 95%CI:2.05-44.60). In ROC curve analysis, the sensitivity was 100% (95%CI: 0.49-1.0) and specificity 77.14% (95%CI:0.60-0.90) for rectal diameter >3 cm. The likelihood ratio was 4.38 (95%CI:2.40-8.0) for the persistence of BBD following treatment.

**Conclusion:**

RD appears to be relevant in the evaluation of children with BBD, not only as a diagnostic tool but also as a predictor of treatment outcome.

## INTRODUCTION

Bladder and Bowel Dysfunction (BBD) is characterized by the presence of Lower Urinary Tract Symptoms (LUTS) associated with Functional Constipation (FC). The physiopathology of BBD lies with anatomical, embryological, and functional interactions(1-3). The presence of a large fecal mass in the rectum that eventually affects bladder capacity, lack of coordination in the pelvic floor muscles/sphincters, central nervous system abnormalities, and cross-organ sensitization between the bladder and the rectum have been implicated in the development of this dysfunction (2, 4–9).

BBD is associated not only with important clinical consequences related to the urinary tract such as urinary infections and possible renal sequelae but also with emotional and behavioral problems (10). The social stigma of fecal and urinary incontinence is a common challenge faced by children with BBD and can lead to issues such as self-esteem, embarrassment, loneliness, a decline in school performance, aggressiveness, and other behavioral issues (10). Early diagnosis and quick management of constipation are considered crucial in achieving an improvement in urinary symptoms (4, 11, 12).

In this context anticholinergics medications can be used to improve symptoms of urinary urgency and daytime incontinence, but exert a negative effect on colonic transit, making FC worse (2). In turn, Parasacral Transcutaneous Electrical Nerve Stimulation (TENS) has been used as an alternative treatment for children with LUTS refractory to behavioral changes in urinary and bowel habits, and this treatment has also been reported to exert an effect on FC, improving urinary and intestinal symptoms in more than 80% of children with BBD when associated with behavioral changes (13).

Measurement of the transverse diameter of the rectum, rectal diameter (RD), has been used as an ancillary tool in the diagnosis of FC in children, with an association already demonstrated between fecal incontinence and enlarged RD (14). RD seems to be a noninvasive exam and a reliable alternative to digital rectal examination in constipated children (15); therefore, it's an easy method and can be particularly useful for diagnosing and treating fecal impaction (16).

Identifying outcome predictors in BBD is essential when planning treatment. According to Hoffman et al., the presence of enuresis in children with an overactive bladder, a clinical condition commonly linked to FC, is a factor associated with a poor response to treatment (17). Furthermore, the persistence of FC itself is associated with the persistence of urinary symptoms (4). Therefore, determining the influence of enlarged RD on treatment outcome can be particularly useful. Indeed, RD is easily obtainable and unrelated to subjective complaints, making it a potential prognostic marker that could help identify the best treatment approach for BBD in children and adolescents.

To the best of our knowledge, no study has yet been conducted to evaluate RD as a predictor of treatment outcome; therefore, the objective of this study was to analyze whether enlarged RD plays any role in the persistence of urinary symptoms and FC in children and adolescents submitted to treatment for BBD. In addition, the study aimed to evaluate the association between RD and response to treatment in children and adolescents with BBD.

## MATERIAL AND METHODS

This cohort study was nested within a randomized clinical trial previously approved by the institute's review board under protocol number 683884517.5.0000.5544 and registered in the Brazilian Clinical Trials Registry as RBR-58c63h (13). The study was conducted by the principles laid out in the Declaration of Helsinki and in compliance with the requirements of Resolution 466/12 of the Brazilian National Health Council (CNS) that regulates research involving human beings. The participation of all the individuals enrolled in the main study was completely voluntary and confidential, with patients being identified exclusively as alphanumerical codes, thus preserving their privacy. All the parents/guardians of the participants in the main study provided written informed consent with guarantees of confidentiality.

### Study setting

The study was conducted between January 2017 and December 2019 in a referral center for the treatment of childhood urinary disorders. Children and adolescents from 5 to 17 years of age diagnosed with BBD and submitted to treatment with behavioral changes associated or not with Parasacral TENS were included. Patients with a diagnosis of anatomic or neurological alterations of the gastrointestinal or urinary tract, such as neurogenic bladder, were excluded from the study.

### Instruments

The Dysfunctional Voiding Scoring System (DVSS) (Supplementary File-1), translated and validated for use in Brazilian Portuguese (18), was used to evaluate urinary symptoms before and following treatment. Bowel symptoms were evaluated using the Rome IV criteria (19) for children and adolescents 4 to 18 years of age. Patients who fulfilled at least two Rome IV criteria were considered constipated.

The Constipation Scoring System (Supplementary file 2) was also used to evaluate the severity of FC (20).

The symptoms referring to items 3, 4, 5, and 7 (feeling of incomplete evacuation, abdominal pain, time in lavatory per attempt, and unsuccessful attempts for evacuation per 24 hours) were also evaluated individually, with scores ≥2 being considered abnormal. Concerning item 6 (use of some type of assistance to defecate), scores ≥1 were considered abnormal. The Bristol Stool Scale was used to analyze the shape of the stool (7).

### Treatment of BBD and rectal diameter measurement

Patients were previously randomized into two groups using sealed envelopes containing four sequential numbers. The control group was submitted to behavioral changes plus sham while the treatment group was submitted to behavioral changes plus Parasacral TENS. Both groups were submitted to treatment for 20 sessions in an intention-to-treat analysis. Behavioral changes consist of the following recommendations: not to wait longer than three hours between voids; to avoid coffee, tea, carbonized drinks, chocolate, and citric fruits during treatment; to void before going to sleep; to drink more fluids during the day (around 5 to 8 X 200 mL glasses/day depending on age); with those complaining of enuresis to avoid drinking fluids at least 2-3 hours before going to sleep. In addition, instructions were also given regarding the appropriate intake of high-fiber foods. However, neither fiber supplements nor specific nutritional diets were recommended for either group. All patients were instructed to sit appropriately on the toilet for 5-10 minutes three times a day after their main meals.

Parasacral TENS consists of placing self-adhesive electrodes on the parasacral region between S2 and S4, with the patient in the ventral decubitus position. The electrodes were connected to an electrical stimulation device with a frequency of 10 Hz and pulse width of 700 μs, three times a week for a total of 20 sessions, with intensity varying according to the patient's tolerance level and without reaching the motor threshold. Sham treatment was performed identically but with no electrical activity, thus functioning as a placebo. None of the patients included in the study used laxatives during treatment.

**Figure 1:**
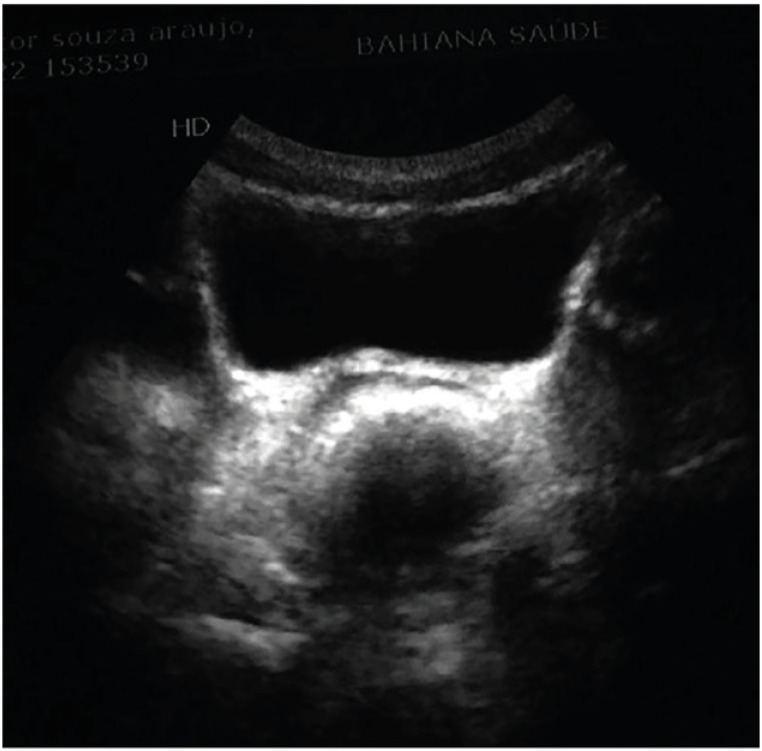
Increased rectal diameter in trans-abdominal ultrasound.

Source: Personal archive.

RD was measured by abdominal ultrasound, performed twice on each patient by the same experienced professional and with the patient in the dorsal decubitus position. A 7-MHz probe was placed on the abdominal wall, two centimeters above the symphysis. To measure RD, bladder capacity should be between 30% and 70% of the expected capacity for age calculated as: (age + 1 × 30) (21). Before the sonographic measurements were obtained, all the children were asked whether they needed to defecate, and the examination was not performed on those who stated that they did or with urge to defecate. Measurement was performed according to the technique established by Klijn et al. (21). Rectal diameters were considered enlarged when ≥3 cm for all patients, as demonstrated in Figure-1, regardless of age, since there is no significant difference in this cut-off in older children and adolescents (21).

### Sample size

This paper is a nested study of a randomized clinical trial (13) which included 40 patients through sample size calculation. We evaluated the area under the ROC curve (AUC) with that number of patients, finding an accuracy of 74.5% with a power of 80% (1- β) and a type I error of 0.05, making it feasible to evaluate the rectal diameter with that number of patients. The sample size was calculated using MedCalc, version 19.5 (MedCalc Software Ltd., Ostend, Belgium; https://www.medcalc.org; 2020).

### Statistical Analysis

Independent categorical variables were described using numbers and percentages and the numerical variables as means and standard deviations (SD) when normally distributed or as medians and interquartile range (IQR) if the distribution was not normal. The normality of the distribution of the variables was tested using the Kolgomorov-Smirnov test.

Clinical characteristics of the study population were described and compared using numerical and categorical variables. Numerical variables were expressed as means and SD or as medians and IQR and compared using Student's t-test or the Mann-Whitney test depending on their distribution. The categorical variables were described using absolute and relative frequencies and compared using Fisher's exact test.

Variables with p-values <0.1 were then selected for inclusion in a multivariate analysis and those subsequently found to have differences with a p-value <0.05 were included in the final multivariate model. Those that were statistically significant in this model were used to analyze the accuracy of the final model. The discrimination ability was analyzed based on the area under the receiver operating characteristic (ROC) curve, adopting a confidence interval of 95% (95%CI). The Hosmer-Lemeshow test was performed to evaluate the goodness of fit.

P-values <0.05 were considered statistically significant. Data for analysis were stored and processed using the Statistical Package for the Social Sciences (SPSS Inc., Chicago, Illinois, USA), version 25.0 for Windows, and MedCalc, version 19.5 (MedCalc Software Ltd., Ostend, Belgium; https://www.medcalc.org; 2020).

## RESULTS

Forty patients with a diagnosis of BBD and a mean age of 8.4 ± 2.8 years were evaluated. Of these, 52.5% were male. Enlarged Rectal Diameter was detected in 15 patients (37.5%) before treatment, with a mean diameter of 3.84 ± 0.6 cm.

### Analysis of the association between the transverse diameter of the rectum before treatment and lower urinary tract symptoms (LUTS), FC and its effect on treatment outcome

Patients with Overactive Bladder and Voiding Postponement were included. No association was found between LUTS and enlarged pretreatment rectal diameter. In addition, there was no association between rectal diameter and the bowel symptoms associated with FC. However, children and adolescents with an enlarged rectal diameter had a significantly higher constipation score (Table-1).

**Table 1:** Association between rectal diameter and the presence of lower urinary tract symptoms and bowel symptoms associated with functional constipation before treatment.

Parameters	Rectal diameter before treatment		p-value
	< 3 cm (n=25)	≥ 3 cm (n=15)	
DVSS, median (IQR)	13 (10-16)	14 (11-17)	0.54†
Urinary urgency, n (%)	25 (100)	14 (93.3)	0.19*
Daytime incontinence, n (%)	23 (92)	12 (80)	0.27*
Holding maneuvers, n (%)	20 (80)	13 (86.7)	0.59*
Daytime urinary frequency, n (%)	14 (56)	9 (60)	0.80*
Nocturia, n (%)	8 (32)	3 (20)	0.33*
Enuresis, n (%)	18 (72)	14 (93.3)	0.10*
Lower urinary tract dysfunction, n (%)	23 (92)	14 (93.3)	0.87*
Constipation score, median (IQR)	9 (6-11)	11 (10-16)	**0.01** †
Feeling of incomplete evacuation, n (%)	13 (52)	7 (46.7)	0.74*
Time in lavatory per attempt >5 minutes, n (%)	14 (56)	12 (80)	0.12*
Assistance to evacuate, n (%)	3 (12)	2 (13.3)	0.90*
Unsuccessful attempt to defecate, n (%)	18 (72)	14 (93.3)	0.10*
Abdominal pain, n (%)	14 (56)	11 (73.3)	0.27*
Bristol Stool Scale: Types 1 and 2, n (%)	12 (48)	5 (33.3)	0.36

IQR = interquartile range; n: number;

* = Fisher's exact test;

† Mann Whitney test.

Evaluation of the measurement of rectal diameter before treatment and of urinary and bowel symptoms following treatment showed that 11 constipated patients (73.3%) with an enlarged pretreatment rectal diameter continued to have bowel dysfunction following the intervention. Of these 11 patients, 9 had been treated with behavioral changes alone and 2 with behavioral changes plus Parasacral TENS. After treatment, these patients also required laxatives and had more severe FC (Table-2).

**Table 2:** Comparison between rectal diameter before treatment and the persistence of lower urinary tract symptoms and functional constipation following treatment.

Parameters	Rectal diameter before treatment		p-value
	<3 cm (n=25)	≥ 3 cm (n=15)	
∆ DVSS, median (IQR)	- 9.4	- 6.2	0.09†
Urinary urgency, n (%)	6 (24%)	8 (53.3)	0.06*
Daytime incontinence, n (%)	8 (32)	7 (46.5)	0.35*
Holding maneuvers, n (%)	5 (20)	7 (46.7)	0.08*
Daytime urinary frequency, n (%)	2 (08)	4 (26.7)	0.11*
Nocturia, n (%)	4 (16)	2 (13.3)	0.82*
Enuresis, n (%)	12 (48)	12 (80)	0.05*
Lower urinary tract dysfunction, n (%)	7 (28)	7 (46.7)	0.23*
Constipation, n (%)	7 (28)	11 (73.3)	**0.005** *
Constipation score, median (IQR)	4 (1.5-6)	9 (4-11)	**0.003** ††
Feeling of incomplete evacuation, n (%)	6 (24)	8 (53.3)	0.06*
Time in lavatory per attempt >5 minutes, n (%)	4 (16)	6 (40)	0.09*
Assistance to evacuate, n (%)	0	2 (13.3)	0.06*
Unsuccessful attempt to defecate, n (%)	6 (24)	14 (93.3)	**< 0.001** *
Abdominal pain, n (%)	7 (28)	7 (46.7)	0.23
Bristol Stool Scale: Types 1 and 2, n (%)	8 (32)	7 (46.7)	0.35
Use of laxatives after treatment, n (%)	6(24)	11 (73.3)	**0.02**

∆ = delta; IQR = interquartile range;

n = number.

* Fisher's exact test;

† Student's t-test;

†† Mann Whitney test.

Patients that underwent different treatments were analyzed as a single group (enlarged RD). Regarding the type of treatment, there was no difference between the two groups (control and treatment groups) concerning to pretreatment rectal diameter or to the frequency of enlarged rectal diameter. In the control group, 9/10 patients (90%) in whom rectal diameter was enlarged continued to have FC following treatment, while 5 patients (50%) in whom rectal diameter was normal had FC following treatment (p=0.51). In the treatment group, 2/5 patients (40%) in whom the rectal diameter was enlarged continued to have FC following treatment (p=0.2). Moreover, 12 (80%) patients with enlarged rectal diameter remained with enuresis (p=0.05).

In the binary regression performed to evaluate whether sex, age or enlarged pretreatment rectal diameter played any role in the persistence of BBD, the model containing enlarged pretreatment rectal diameter was statistically significant [X 2 (1) 9.66 = 0.002; R2 Nagelkerke = 0.368]. Enlarged pretreatment rectal diameter was the only independent predictor (OR=9.56; 95%CI: 2.05-44.60) in the model found to affect the persistence of BBD following treatment.

### The accuracy of rectal diameter as a predictor of response to treatment

**Figure 2:**
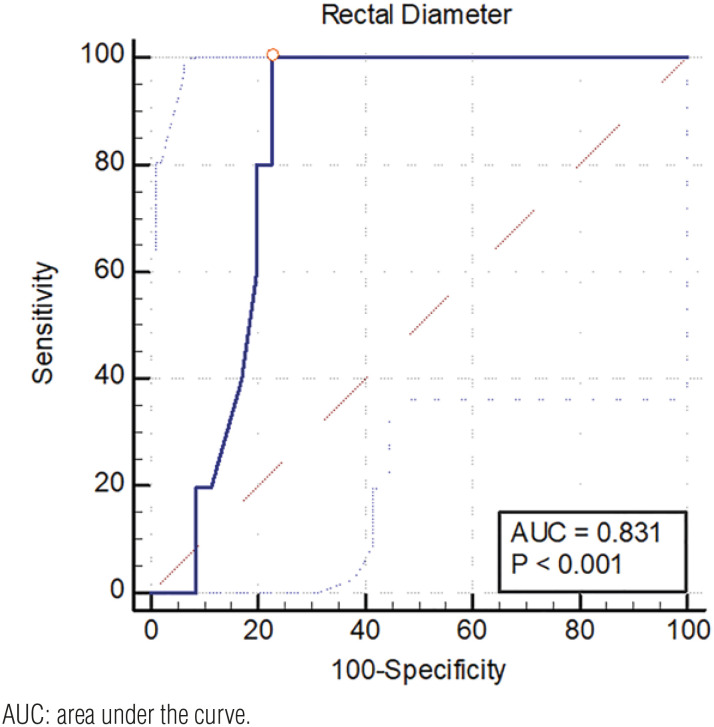
The area under the receiver operating characteristic (ROC) curve for rectal diameter as a predictor of treatment outcome in children with bladder and bowel dysfunction. AUC: area under the curve.

Rectal diameter was included in the model to generate the ROC Curve (Figure-2). Rectal diameter >3.09 cm had an area under the curve of 0.83 (95%CI: 0.68-0.91) (p<0.0001) for predicting the persistence of constipation and urinary incontinence following treatment. In the association analysis, for rectal diameter >3.09 cm sensitivity was 100% (95%CI: 0.48 - 1.0) and specificity 77.14% (95%CI: 0.60 - 0.90). The likelihood ratio was 4.38 (95%CI: 2.40 - 8.0) for the persistence of constipation and urinary incontinence following TENS treatment.

## DISCUSSION

Previous studies have described rectal diameter as a tool for diagnosing FC since this ultrasound measurement is enlarged in constipated children compared to children with no bowel dysfunction (14, 21, 22). Nevertheless, evaluation of the association between pretreatment rectal diameter and the persistence of BBD following treatment showed that the children in whom rectal diameter was greater were more likely to remain constipated and less likely to achieve a successful outcome. This finding is important since, in our perspective, no studies in the literature have evaluated the value of rectal diameter in predicting treatment outcomes in children and adolescents with BBD. Our study also used an objective finding to evaluate the response to treatment, less subject to bias.

The present study found no association between enlarged pretreatment rectal diameter and LUTS, the severity of LUTS or bowel complaints. However, an association was found between this measurement and higher constipation scores, with the rectal diameter being greater in children with more severe constipation. Therefore, the use of rectal diameter could increase the accuracy of a diagnosis of BBD.

There is still controversy in the literature regarding the use of rectal diameter as a tool with which to diagnose FC in children, with a diagnosis of this dysfunction remaining essentially of a clinical nature and based on the Rome IV criteria. However, an association between rectal diameter >3 cm and the presence of FC has been reported, particularly when fecal impaction is present, and rectal diameter is greater in constipated children with fecal incontinence compared to children without FC(14, 16, 23). On the other hand, there are controversies concerning the use of the Rome criteria for diagnosing FC, with differences between patients and physicians regarding what should be considered FC (24). Rectal diameter may be particularly useful in those patients meeting only one Rome criterion as this measurement could eliminate the need for digital rectal examination in these cases.

Although there are studies on rectal diameter as a tool for diagnosing FC, its prognostic value has never been investigated. Therefore, by evaluating its predictive accuracy based on the ROC curve, the present study showed that this ultrasound measurement might have prognostic value in predicting treatment outcomes in children and adolescents with BBD. This study also showed that constipated patients with enlarged rectal diameters still required laxatives after treatment. This finding suggests that more aggressive management consisting of behavioral changes, electrical therapy, and laxatives is needed in children with an enlarged rectal diameter.

This study opens new perspectives to individualize patients' treatment based on rectal diameter. If the rectal diameter has a normal value, we could be less aggressive. However, a combination therapy (e.g., parassacral TENS plus laxatives) could be considered in the case of enlarged rectal diameter.

Despite laxatives being the first line for the treatment of functional constipation, behavior changes have still been used in some cases (4). We emphasize that this study is nested in a randomized clinical trial that evaluated the efficacy of the association between parasacral TENS and behavioral changes without using laxatives (13). Therefore, some patients only experienced behavioral changes because they were part of this randomized clinical trial. Some children may not tolerate the laxative's untasteful flavor and adverse effects, like abdominal pain and diarrhea. Furthermore, after intensive initial medical and behavioral treatment, about 40% of patients still were constipated at 1 year of follow-up and, 25% continue to have severe constipation during puberty (25). In this way, new therapies have been designed for the management of bowel dysfunction, such as neuromodulation.

Our results suggest that children and adolescents with less severe constipation may not require laxatives, making them useful for those with more severe constipation that doesn't respond to initial treatment. Those patients could be the ones with enlarged rectal diameters in the pre-treatment evaluation.

Moreover, we found a marginal association between maintained enuresis and increased rectal diameter. An association between enuresis and rectal diameter has already been seen by Jansson et al. (26), but the lack of larger sample size makes an underpowered analysis. Hoffman et al. (17) showed that enuresis was the only symptom associated with a poor outcome after Parasacral TENS in children with OAB, therefore, this relationship could exist making an impact on rectal diameter assessment beyond FC. In this way, the rectal diameter can also be useful in the evaluation of the enuretic patient. Thus, our findings reinforce the idea that rectal diameter should be incorporated into the evaluation of children with LUTS and FC, not only as a diagnostic tool but also as a predictor of response to treatment. Regarding our study that hadn't found an association between rectal diameter and other urinary symptoms, our sample size could have influenced this result, since we had focused on children with BBD, not only in those with LUTS. Further studies should be done to elucidate the association between rectal diameter and LUTS.

As shown in Table-1, there was no association between rectal diameter and bowel symptoms associated with FC. However, children and adolescents with increased rectal diameter had a significantly higher constipation score. This finding suggests that there may not be a clear association between the diagnosis of constipation based on Rome IV and its assessment of the constipation score, since some of the signs and symptoms surveyed differ between these two instruments. This is a gap that is now being studied by other researchers in our group.

Nonetheless, there are certain limitations associated with the study that must be mentioned. The existence of two treatment groups and the size of the sample may have been confounding factors, making some analyses underpowered. However, no association was found between the type of treatment and pretreatment rectal diameter measurement or with the frequency of enlarged rectal diameter. This could be a consequence of the randomization of the initial trial. Further studies are required to confirm the hypothesis tested here that rectal diameter is a predictor of treatment outcome in children and adolescents with BBD.

Another limitation is that RD was considered enlarged when ≥3 cm for all patients, regardless of age. To our knowledge, there is no significant difference in this cut-off in older children and adolescents, according to Klijn et al. (21). But we think that the compliance of the rectum could change with age and consequently, change this cut-off. Further studies evaluating this question should be done to elucidate this gap in literature. As no patient in our study utilized laxatives throughout therapy, we are aware that our results cannot be generalized to encompass alternative treatment modalities.

## CONCLUSIONS

Enlarged rectal diameter (>3cm) appears to be relevant in the evaluation of children with BBD, not only as a diagnostic tool but also as a predictor of treatment outcome. However, more research should be conducted to confirm this finding, which will aid in evaluating the patient's treatment.

## Data Availability

The data supporting this study's findings are available on request from the corresponding author.

## Abbreviations

95%CI =: 95% Confidence Interval
BBD =: Bladder and Bowel Dysfunction
DVSS =: Dysfunctional Voiding Scoring System
FC =: Functional Constipation
IQR =: Interquartile Range
LUTS =: Lower Urinary Tract Symptoms
OR =: Odds Ratio
RD =: Rectal Diameter
ROC curve =: Receiver Operating Characteristic Curve
SD =: Standard Deviation
TENS =: Transcutaneous Electrical Nerve Stimulation

## APPENDIX:

### Supplementary File 1 Dysfunctional Voiding Scoring System (DVSS) for children.

**Table:**

Over the last month	Almost never	Less than half the time	About half the time	Almost every time	Not available
1. I have had wet clothes ot wet underwear during the day.	0	1	2	3	NA
2. When I wet myself, my underwear is soaked.	0	1	2	3	NA
3. I miss having a bowel movement every day.	0	1	2	3	NA
4. I have to push for my bowel movements to come out.	0	1	2	3	NA
5. I only go to the bathroom one or two times each day.	0	1	2	3	NA
6. I can hold onto my pee by crossing my legs, squatting or doing the "pee dance".	0	1	2	3	NA
7. When I have to pee, I cannot wait.					
8. I have to push to pee.	0	1	2	3	NA
9. When I pee it hurts.	0	1	2	3	NA
10. Parents to answer. Has your child experienced something stressful like the example below?	NO (0)			YES (3)	
TOTAL					

### Supplementary File 2 Constipation Score for children.

**Table:**

**Frequency of bowel movements**			**Minutes in the toilet to defecate**		
	0.	1-2 times per 1-2 days		0.	Less than 5 minutes
	1.	2 times per week		1.	5-10 minutes
	2.	Once per week		2.	10-20 minutes
	3.	Less than once per week		3.	20-30 minutes
	4.	Less than once per month		4.	More than 30 minutes
**Painful evacuation effort**			**Type of assistance**		
	0.	Never		0.	No assistance
	I.	Rarely		1.	Stimulative laxatives
	2.	Sometimes		2.	Digital assistance or enema
	3,	Usually			
	4.	Always			
**Feeling incomplete evacuation**			**Unsuccessful attempts for evacuation per 24 hours**		
	0.	Never		0.	Never
	1.	Rarely		1.	1-3
	2.	Sometimes		2.	3-6
	3.	Usually		3.	6-9
	4.	Always		4.	More than 9
**Abdominal pain**			**Duration of constipation**		
	0.	Never		0.	Less than 1 year
	1.	Rarely		1.	1-3 years
	2.	Sometimes		2.	3-5 years
	3.	Usually		3.	5-7 years
	4.	Always		4.	More than 7 years
